# Pathophysiological Roles of Oxidative Stress and the Translational Potential of Antioxidant Therapy in Retinal Vein Occlusion

**DOI:** 10.3390/antiox15030338

**Published:** 2026-03-07

**Authors:** Hidetaka Noma, Tatsuya Mimura

**Affiliations:** 1 Department of Ophthalmology, Tokyo Medical University Ibaraki Medical Center, Chuo, Amimachi, Inashiki-gun 300-0395, Ibaraki, Japan; 2 Department of Ophthalmology, Tsurumi University School of Dental Medicine, Tsurumi, Tsurumi-ku, Yokohama 230-8501, Kanagawa, Japan

**Keywords:** retinal vein occlusion, oxidative stress, antioxidants, reactive oxygen species, blood–retinal barrier, vascular endothelial growth factor

## Abstract

Retinal vein occlusion (RVO) is the second most common retinal vascular disorder after diabetic retinopathy and represents a major cause of visual impairment worldwide. In addition to venous congestion, endothelial dysfunction, and inflammation, accumulating evidence indicates that oxidative stress plays a pivotal role in the pathogenesis of RVO. The excessive production of reactive oxygen species (ROS) during ischemia–reperfusion injury induces endothelial damage, disruption of the blood–retinal barrier, and upregulation of inflammatory cytokines and vascular endothelial growth factor (VEGF), thereby contributing to macular edema and progressive visual dysfunction. This review summarizes current knowledge from both experimental and clinical studies regarding the mechanisms of oxidative stress generation in RVO and its underlying molecular pathways, highlighting the pathological consequences of impaired antioxidant defense systems. We further review reported alterations in oxidative stress markers and antioxidant factors in serum, aqueous humor, and vitreous fluid, and discuss their potential associations with disease activity and visual prognosis. In addition, the interplay between oxidative stress and current standard treatments, including anti-VEGF therapy and corticosteroids, is discussed, together with the translational potential of antioxidant strategies such as polyphenols, vitamins, and Nrf2 pathway activators. At the same time, we address critical challenges limiting clinical application, including insufficient interventional evidence, the lack of validated biomarkers, and uncertainties regarding optimal timing of antioxidant intervention. By providing a comprehensive overview of oxidative stress in RVO, this review aims to identify emerging therapeutic targets and opportunities for personalized treatment approaches, and to outline future research directions toward improving long-term visual outcomes in patients with RVO.

## 1. Introduction

Retinal vein occlusion (RVO) is the second most common retinal vascular disease after diabetic retinopathy and represents a major cause of visual impairment worldwide. It leads to vision loss primarily through retinal hemorrhage, macular edema, and retinal ischemia [1]. Clinically, RVO is classified into central retinal vein occlusion (CRVO) and branch retinal vein occlusion (BRVO), which differ in disease severity, prognosis, and therapeutic response [2].

The pathogenesis of RVO is multifactorial and involves venous stasis, endothelial dysfunction, inflammatory responses, and ischemia–reperfusion injury [3]. In recent years, oxidative stress has emerged as a common pathogenic denominator underlying these processes. Excessive production of reactive oxygen species (ROS) under hypoxic conditions has been implicated in the disruption of the blood–retinal barrier (BRB), increased vascular permeability, and subsequent neuroretinal damage [4].

In addition to inducing mitochondrial dysfunction and cellular apoptosis, oxidative stress is closely linked to the upregulation of vascular endothelial growth factor (VEGF), which plays a central role in the development of macular edema in RVO [5]. Currently, intravitreal anti-VEGF therapy constitutes the mainstay of treatment for RVO-associated macular edema. However, considerable variability in treatment response, frequent recurrence, and incomplete functional recovery remain significant clinical challenges. These limitations suggest the involvement of VEGF-independent mechanisms, particularly oxidative and inflammatory pathways, in RVO pathophysiology [6].

Against this background, antioxidant strategies have attracted increasing attention as a potential therapeutic approach for RVO. Experimental studies have demonstrated that mitochondria-targeted antioxidants, inhibition of nicotinamide adenine dinucleotide phosphate (NADPH) oxidase, and activation of the nuclear factor erythroid 2-related factor 2 (Nrf2) pathway—an essential regulator of endogenous antioxidant defense—can attenuate retinal ischemia–reperfusion injury and vascular dysfunction [7]. Nevertheless, clinical evidence supporting the application of these antioxidant approaches in RVO remains limited, and a comprehensive synthesis of existing data is still lacking.

Several previous reviews have addressed oxidative stress and antioxidant strategies in retinal diseases. However, many of these reviews have focused broadly on chronic retinal degenerative disorders or have discussed oxidative mechanisms without specifically addressing the unique pathophysiological features of RVO. In contrast, the present review specifically centers on RVO as an acute thrombo-ischemic retinal disorder and integrates recent advances in mitochondrial ROS biology, pathway-specific modulators, and translational challenges that may explain discrepancies between experimental and clinical outcomes.

This article was conducted as a narrative review with a structured literature search strategy. Electronic databases including PubMed, Web of Science, and Scopus were searched for studies published up to February 2026. The following keywords were used in various combinations: “retinal vein occlusion”, “oxidative stress”, “reactive oxygen species”, “antioxidant”, “ischemia–reperfusion”, “mitochondrial dysfunction”, and “anti-VEGF resistance.” Both experimental and clinical studies were considered. This review aims to provide a mechanistic and translational synthesis rather than a formal systematic meta-analysis.

**Figure 1:**
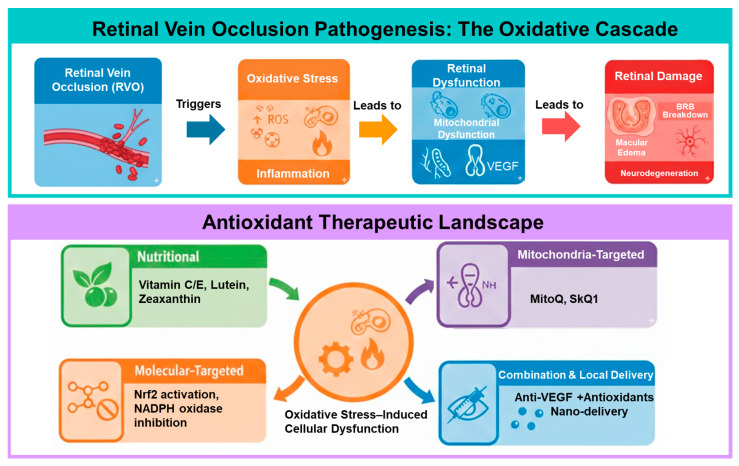
Oxidative stress-related pathophysiology and therapeutic targets in RVO. Retinal vein occlusion (RVO) induces retinal ischemia and hypoxia, leading to excessive production of reactive oxygen species (ROS) and mitochondrial dysfunction in retinal endothelial cells, retinal pigment epithelium (RPE), and neurons. Oxidative stress activates inflammatory signaling pathways and upregulates vascular endothelial growth factor (VEGF), resulting in blood–retinal barrier (BRB) breakdown, increased vascular permeability, and macular edema. Antioxidant interventions targeting mitochondrial ROS, redox imbalance, and oxidative–inflammatory cascades may attenuate endothelial injury, suppress VEGF overexpression, and preserve retinal structure and function. These strategies are expected to complement current anti-VEGF therapies and may offer additional benefits, particularly when applied at appropriate disease stages and patient subtypes (e.g., central vs. branch RVO). Abbreviations: RVO, retinal vein occlusion; ROS, reactive oxygen species; VEGF, vascular endothelial growth factor; Nrf2, nuclear factor erythroid 2-related factor 2; NADPH, nicotinamide adenine dinucleotide phosphate (reduced form); MitoQ, mitoquinone (mitochondria-targeted ubiquinone); SkQ1, 10-(6′-plastoquinonyl) decyltriphenylphosphonium.

Therefore, this review aims to systematically summarize current basic and clinical evidence regarding the role of oxidative stress in RVO and to discuss the therapeutic potential of antioxidant interventions. To our knowledge, this is the first review to systematically integrate RVO-specific oxidative stress mechanisms with emerging pathway-modulating therapeutic strategies and a critical appraisal of translational limitations. Particular emphasis is placed on cell-specific pathogenic mechanisms, molecular targets, associations with retinal imaging findings, and future therapeutic strategies and research directions. An overview of oxidative stress-based pathophysiology and potential therapeutic intervention points in RVO is illustrated in Figure 1.

### 1.1. Epidemiology and Clinical Significance of Retinal Vein Occlusion (Central and Branch Types)

Large-scale systematic reviews and population-based studies have demonstrated that the prevalence and incidence of RVO increase markedly with age, affecting tens of millions of individuals worldwide. Epidemiological analyses consistently identify hypertension as the strongest risk factor for RVO, as evidenced by meta-analyses and data from the U.S. National Health and Nutrition Examination Survey (NHANES). Clinically, it is important to distinguish between CRVO and BRVO, as these entities differ in patient characteristics, disease severity, and visual prognosis [8,9].

### 1.2. Multifactorial Pathogenesis of RVO: Vascular, Metabolic, and Inflammatory Components

The pathogenesis of RVO is multifactorial, involving a complex interplay between local hemodynamic disturbances (venous stasis and reduced blood flow), hematologic and vascular factors (prothrombotic tendency, platelet activation, and elevated homocysteine levels), systemic metabolic conditions (hypertension, dyslipidemia, and diabetes mellitus), and intraocular as well as systemic inflammatory responses. Endothelial dysfunction and disruption of the BRB alter interactions between circulating blood components and retinal tissue, thereby promoting coagulation activation and amplification of local inflammation at sites of blood flow stagnation. These interrelated vascular, coagulative, and inflammatory mechanisms form the fundamental basis for current clinical management strategies, including anti-VEGF therapy, intravitreal corticosteroids, and consideration of antithrombotic approaches [10,11,12].

### 1.3. Evidence for the Involvement of Oxidative Stress in RVO and the Potential Significance of Antioxidant Therapy

Accumulating evidence over the past decade indicates that oxidative stress plays a critical role in the onset and progression of RVO. Clinically, increased levels of oxidative damage markers—such as total oxidant status/total antioxidant capacity imbalance and lipid peroxidation products—have been reported in the aqueous humor and vitreous fluid of eyes with RVO. These findings suggest that enhanced local oxidative burden contributes to increased vascular permeability, endothelial dysfunction, and induction of inflammatory mediators within the retinal microenvironment.

**Figure 2:**
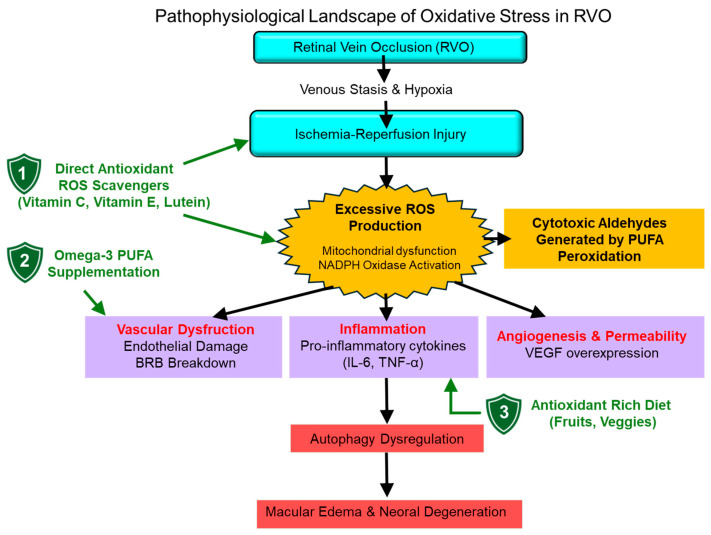
Pathophysiological Landscape of Oxidative Stress in Retinal Vein Occlusion (RVO). The obstruction of retinal veins triggers a cascade starting with venous stasis and hypoxia, leading to ischemia–reperfusion injury and the burst of reactive oxygen species (ROS). This oxidative environment induces the peroxidation of polyunsaturated fatty acids (PUFAs), particularly DHA, which is highly concentrated in the retina. The resulting chronic oxidative load promotes a “vicious cycle” involving: (1) Endothelial dysfunction and Blood–Retinal Barrier (BRB) breakdown; (2) Upregulation of VEGF and pro-inflammatory cytokines; and (3) Autophagy dysregulation leading to photoreceptor and RPE cell death. Antioxidant therapies (green indicators) aim to mitigate these processes by direct ROS scavenging, stabilizing vascular membranes, and restoring homeostatic autophagy, thereby preventing macular edema and permanent vision loss.

Experimental studies further demonstrate that retinal ischemia–reperfusion injury and venous stasis promote excessive ROS production, leading to activation of molecular pathways implicated in cell death and vascular injury, including mitogen-activated protein kinase (MAPK), nuclear factor kappa-light-chain-enhancer of activated B cells (NF-κB), and NADPH oxidase-dependent signaling. Collectively, these data support the concept that modulation of oxidative stress represents a promising therapeutic strategy for RVO [13]. An overview of oxidative stress-related mechanisms in RVO is illustrated in Figure 2.

Based on these considerations, RVO management may benefit from therapeutic approaches that extend beyond conventional hemodynamic and anti-VEGF-centered strategies to include interventions targeting oxidative stress, either locally or systemically. Antioxidants and free radical scavengers, such as edaravone in experimental models, have shown potential for retinal cell protection and improvement of macular edema and vascular permeability. However, further well-designed preclinical and clinical studies are required to translate these findings into clinical practice [7,14].

## 2. Retinal Vulnerability to Oxidative Stress

The retina is a tissue with exceptionally high physiological oxygen consumption and metabolic activity, rendering it highly susceptible to even subtle disturbances in redox homeostasis, which can readily translate into tissue injury [7]. Oxidative stress has therefore been widely implicated as a fundamental pathogenic mechanism across a broad spectrum of retinal diseases [14].

### 2.1. Imbalance Between Oxygen Supply and Demand Induced by Reduced Retinal Blood Flow and Venous Stasis

In RVO, venous stasis and localized reductions in retinal blood flow lead to an imbalance between oxygen supply and cellular oxygen consumption. This ischemia–reperfusion-like microenvironment promotes a rapid surge in ROS, resulting in functional impairment and cell death—via apoptosis and necrosis—of endothelial cells, glial cells, and neurons, as demonstrated in animal models and molecular studies [14]. These processes contribute to disruption of the BRB and increased vascular permeability, thereby facilitating VEGF upregulation and the progression of macular edema and visual dysfunction [6,14].

### 2.2. High Metabolic Activity, Lipid-Rich Retinal Tissue, and Susceptibility to ROS

Clinical studies have reported significantly elevated serum levels of oxidative stress markers, including malondialdehyde (MDA), 8-hydroxy-2′-deoxyguanosine (8-OHdG), and hydrogen peroxide, accompanied by reduced activities of antioxidant enzymes such as Superoxide dismutase (SOD) and catalase in patients with RVO [15]. These findings reflect an increased systemic oxidative burden and provide clinical evidence supporting oxidative stress as an integral component of RVO pathophysiology.

In addition, hematological alterations contribute to the generation and amplification of oxidative stress in RVO. Enhanced ROS production and lipid peroxidation have been observed in erythrocytes from patients with RVO, which are associated with reduced erythrocyte deformability and increased blood viscosity, thereby exacerbating microcirculatory impairment [16]. During thrombus formation, activated platelets and neutrophils serve as local sources of ROS, and neutrophil extracellular traps (NETs) may further contribute to oxidative stress, potentially facilitating thrombus formation and persistence [17].

The intrinsic composition of retinal tissue further increases its vulnerability to oxidative injury. The outer retina contains photoreceptor outer segment disk membranes enriched in Polyunsaturated fatty acid (PUFA), particularly docosahexaenoic acid (DHA), which are highly susceptible to oxidation under conditions of light exposure and ROS generation [18]. DHA is present at high concentrations in the retina, and under oxidative conditions readily undergoes lipid peroxidation, generating cytotoxic lipid peroxides that can induce cellular damage [19]. Consequently, in the retina—where high oxygen consumption and light exposure coexist—oxidative modification products may accumulate, impairing photoreceptor and RPE function.

### 2.3. Phototoxicity and Oxidative Modifications of Blood Components Associated with Hemodynamic Disturbance

Finally, the interplay between hemodynamic disturbance, enhanced phototoxicity, and oxidative modification of blood components warrants consideration. Reduced retinal blood flow induces fluctuations in local oxygen tension, and abrupt restoration of oxygen supply during reperfusion or thrombolysis predisposes to excessive ROS generation [20]. Moreover, within a procoagulant environment, oxidative modifications driven by hemoglobin oxidation, ROS derived from platelets and neutrophils, and myeloperoxidase (MPO) activity may progress, leading to dysfunction of erythrocyte membranes, vascular walls, and plasma proteins [21].

In the retina—a highly sensitive tissue—these blood flow- and blood component-derived oxidative modifications can rapidly translate into tissue injury, inflammatory responses, and increased vascular permeability. Accordingly, oxidative stress is considered to play a central role in the pathogenesis of RVO [7,14,22].

Taken together, the post-RVO retina is particularly vulnerable to oxidative stress at three interrelated levels: (1) hemodynamic alterations that predispose to imbalance between oxygen supply and demand; (2) a highly metabolic, PUFA-rich tissue environment that is susceptible to ROS-induced lipid peroxidation; and (3) local amplification of oxidative stress originating from blood components, including erythrocytes, platelets, and neutrophils. These considerations provide a strong biological rationale for antioxidant interventions and targeting of specific ROS sources as therapeutic strategies aimed at preserving visual function in RVO [7,14].

## 3. Sources of Oxidative Stress in RVO

The pathogenesis of RVO involves a complex interplay of hemodynamic disruption, alterations in blood components, and metabolic dysfunction within retinal cells, with increased oxidative stress positioned as a central pathogenic mechanism. Importantly, oxidative stress in RVO does not arise from a single source; rather, it is amplified through reciprocal interactions between systemic factors related to blood flow and blood constituents, and local factors operating within retinal tissue [7,14]. Below, the major sources of oxidative stress in RVO are discussed.

### 3.1. Oxidative Stress Derived from Hemodynamic Disturbance and Blood Components

#### 3.1.1. Hypoxia and Ischemia–Reperfusion Injury Induced by Venous Stasis

In RVO, venous obstruction leads to stagnation of retinal circulation, resulting in reduced perfusion pressure within the capillary bed and subsequent tissue hypoxia. Under hypoxic conditions, the efficiency of the mitochondrial electron transport chain is impaired, promoting increased production of ROS, particularly superoxide. Furthermore, during spontaneous recanalization, collateral vessel formation, or restoration of blood flow following therapeutic intervention, an ischemia–reperfusion-like injury occurs, characterized by an abrupt and excessive burst of ROS generation [14,21].

Reperfusion-associated ROS contribute to endothelial dysfunction, the breakdown of the BRB, and upregulation of VEGF, thereby facilitating the progression of macular edema and ischemic retinal changes. Consistent with this concept, antioxidant interventions have been shown to attenuate reperfusion injury in animal and retinal ischemia models, supporting the pathological relevance of ischemia–reperfusion-like conditions in RVO [7].

#### 3.1.2. ROS Production by Activated Platelets and Leukocytes

Thrombus formation and blood flow stasis in RVO are accompanied by activation of platelets and leukocytes, particularly neutrophils. Activated platelets generate ROS via NADPH oxidase, contributing to endothelial injury and stabilization of thrombi [23]. Neutrophils further amplify the oxidative milieu through MPO activity and formation of NETs, thereby exacerbating local inflammation and vascular damage [24].

Recent studies have reported elevated levels of NET-associated markers in patients with RVO, highlighting a pathophysiological axis in which oxidative stress, immune activation, and coagulation are tightly interconnected [14]. In a highly sensitive tissue such as the retina, ROS derived from blood components may induce substantial tissue injury even in the setting of relatively modest circulatory disturbances.

#### 3.1.3. Hematological Risk Factors: Hyperhomocysteinemia and Dyslipidemia

Hyperhomocysteinemia, a well-established risk factor for RVO, is a potent inducer of oxidative stress and promotes endothelial dysfunction and thrombogenesis [25]. Homocysteine directly enhances ROS production while simultaneously reducing the bioavailability of nitric oxide (NO), thereby impairing vasodilatory capacity [26]. In addition, oxidized low-density lipoprotein (LDL) in dyslipidemia exerts cytotoxic effects on vascular endothelium and contributes to chronic oxidative burden [27].

These systemic hematological factors may prime the retinal microenvironment toward a pro-oxidative state, thereby accelerating disease progression once venous occlusion occurs [7].

### 3.2. Oxidative Stress Derived from Intracellular and Local Retinal Factors

#### 3.2.1. Mitochondrial Dysfunction in Retinal Pigment Epithelium (RPE), Capillary Endothelial Cells, and Photoreceptors

RPE cells, retinal capillary endothelial cells, and photoreceptors are highly metabolically active and rely heavily on mitochondrial energy production. Under conditions of hypoxia and inflammation induced by RVO, mitochondrial function becomes compromised, leading to increased electron leakage and excessive ROS generation [28]. Mitochondria-derived ROS act not only as cytotoxic molecules but also as intracellular signaling mediators that trigger inflammatory responses and apoptosis, ultimately contributing to disruption of the retinal neurovascular unit.

#### 3.2.2. Cytokine-Induced Activation of NADPH Oxidase and Sustained ROS Production

In RVO, elevated intraocular levels of inflammatory cytokines—including tumor necrosis factor alpha (TNF-α), Tumor necrosis factor alpha (IL-6), and monocyte chemoattractant protein-1 (MCP-1)—have been reported in both retinal tissue and vitreous fluid. These cytokines activate NADPH oxidase, resulting in sustained ROS production [29]. NADPH oxidase (NOX)-derived ROS contribute to increased vascular permeability and endothelial injury, thereby promoting macular edema. Moreover, persistent oxidative signaling may partly underlie resistance to anti-VEGF therapy, highlighting the relevance of VEGF-independent oxidative pathways.

#### 3.2.3. Reduced Antioxidant Capacity Associated with Aging and Comorbidities

Aging, the strongest risk factor for RVO, is accompanied by a decline in endogenous antioxidant defense systems, including SOD, catalase, and glutathione (GSH)-dependent pathways. Comorbid conditions such as diabetes and hypertension further establish a state of chronic oxidative stress, reducing tissue resilience at the onset of RVO [30]. In this context, oxidative stress induced by venous occlusion may not be adequately neutralized, leading to amplification of retinal tissue damage.

### 3.3. Summary of Sources of Oxidative Stress in RVO

Collectively, oxidative stress in RVO arises from layered contributions of exogenous factors related to hemodynamics and blood components, as well as endogenous factors originating from retinal cells themselves. This multifactorial oxidative milieu provides a strong theoretical rationale for antioxidant therapies and strategies targeting specific ROS sources as adjunctive treatments in RVO.

## 4. Molecular and Cellular Mechanisms by Which Oxidative Stress Drives RVO Pathology

**Figure 3:**
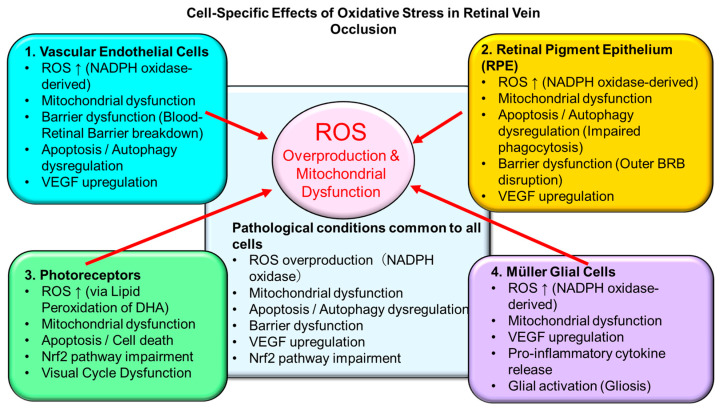
Cell-Specific Effects of Oxidative Stress in Retinal Vein Occlusion Oxidative stress affects multiple retinal cell types, including vascular endothelial cells, retinal pigment epithelium (RPE), photoreceptors, and Müller glial cells. Excessive reactive oxygen species (ROS), derived mainly from NADPH oxidase activation and mitochondrial dysfunction, induce apoptosis, autophagy dysregulation, barrier breakdown, and VEGF upregulation. Impairment of the Nrf2-mediated antioxidant pathway further exacerbates oxidative damage, contributing to blood–retinal barrier disruption and retinal dysfunction in RVO.

In RVO, oxidative stress is not merely a secondary consequence of impaired blood flow but functions as a central pathogenic driver that integrates endothelial dysfunction, inflammation, coagulation abnormalities, and retinal cell death. Excessive ROS exert multifaceted molecular and cellular effects on both retinal vasculature and neural tissue, thereby contributing to disease progression and chronicity in RVO [7,14]. Figure 3 illustrates the cell type-specific impacts of oxidative stress in RVO.

### 4.1. Endothelial Dysfunction and Disruption of the BRB

Retinal vascular endothelial cells form the BRB and play a pivotal role in maintaining retinal homeostasis. Under hypoxic and reperfusion conditions associated with RVO, excessive ROS derived from mitochondria and NOX are generated, leading to endothelial dysfunction [29].

Oxidative stress induces downregulation and structural alteration of tight junction proteins, including occludin, claudin-5, and zonula occludens-1 (ZO-1), thereby compromising the selective permeability of the BRB. This disruption permits leakage of plasma components into the retinal parenchyma and establishes the molecular basis for macular edema formation [31]. Moreover, ROS potentiate VEGF signaling and enhance vascular permeability through both VEGF-dependent and -independent pathways. Clinical observations demonstrating correlations between oxidative stress markers and intraocular VEGF levels in RVO further support oxidative stress as an upstream regulator of BRB breakdown [14].

### 4.2. Lipid Peroxidation and Oxidative Modification of Proteins in Vascular and Coagulation Abnormalities

Oxidative stress in RVO robustly induces lipid peroxidation in endothelial membranes and blood components. Cell membranes enriched in PUFA are particularly susceptible, leading to accumulation of lipid peroxidation products such as MDA and 4-hydroxynonenal (4-HNE). These reactive aldehydes impair membrane fluidity and disrupt the function of receptors and ion channels [32].

In addition, ROS mediate oxidative modification of endothelial proteins, plasma proteins, and coagulation factors, thereby disrupting the homeostasis of coagulation and fibrinolytic systems. Oxidatively modified fibrinogen and platelet membrane proteins exhibit enhanced thrombogenicity, further aggravating local blood stasis and hypoxia within the occluded retinal vein [23]. This positive feedback loop between oxidative stress and coagulation abnormalities constitutes a key molecular basis for the progressive and persistent nature of RVO.

### 4.3. Crosstalk with the Complement System and Inflammatory Responses: The Inflammation–Oxidative Stress Loop

**Figure 4:**
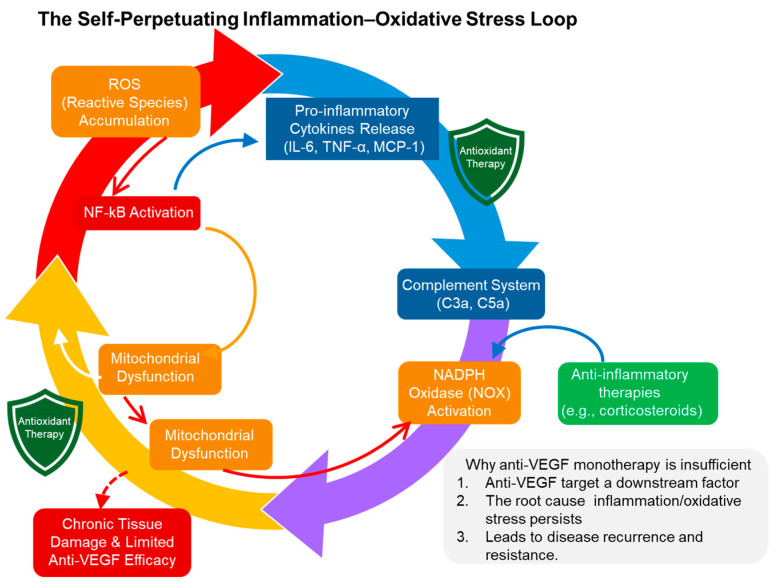
The Self-Perpetuating Inflammation–Oxidative Stress Loop in Retinal Pathophysiology. This conceptual diagram illustrates the vicious cycle driven by the interplay between oxidative stress and chronic inflammation. Reactive Oxygen Species (ROS) accumulation triggers the activation of the master inflammatory regulator NF-κB, leading to the release of pro-inflammatory cytokines (e.g., IL-6, TNF-α, MCP-1) and activation of the complement system. These inflammatory mediators, in turn, activate NADPH oxidase (NOX) and induce mitochondrial dysfunction, causing further ROS production and reinforcing the loop. This chronic feedback loop explains why anti-VEGF monotherapy often shows limited efficacy; while anti-VEGF agents target a downstream consequence (angiogenesis and permeability), the underlying “root cause”—persistent inflammation and oxidative stress—remains unaddressed, leading to disease recurrence and resistance. Synergistic intervention using both antioxidant and anti-inflammatory therapies is proposed to break this cycle and provide robust, long-lasting neurovascular protection.

Oxidative stress is tightly coupled with inflammatory signaling and complement activation, forming a self-amplifying inflammation–oxidative stress loop. As depicted in Figure 4, ROS activate redox-sensitive transcription factors such as NF-κB and activator protein-1 (AP-1), thereby promoting the expression of inflammatory cytokines including TNF-α, IL-6, and MCP-1. In turn, these mediators stimulate NOX activity, leading to sustained ROS production [29].

Furthermore, oxidatively modified cell membranes and proteins serve as triggers for complement activation. Formation of the membrane attack complex (MAC) amplifies endothelial injury and vascular dysfunction [33]. This interplay among complement activation, oxidative stress, and inflammation has been implicated in chronic inflammatory states and may contribute to resistance to anti-VEGF therapy in RVO [7].

### 4.4. Retinal Damage via Dysregulation of Apoptosis, Necrosis, and Autophagy

Excessive oxidative stress disrupts the regulation of cell death pathways in retinal tissue. Moderate ROS levels induce apoptosis through mitochondrial pathways, characterized by cytochrome c release and caspase activation. In contrast, acute and excessive ROS surges lead to ATP depletion and necrosis-like cell death, thereby expanding inflammatory damage to surrounding tissue [34].

Recent studies have also highlighted the role of dysregulated autophagy in ischemic retinal diseases, including RVO. Physiological autophagy exerts cytoprotective effects by removing oxidatively damaged mitochondria and proteins; however, insufficient or excessive autophagic activity may exacerbate cell death. Oxidative stress is a key regulator of autophagy, and its dysregulation likely contributes to progressive neurodegeneration and vascular damage in RVO [35].

### 4.5. Summary of Molecular and Cellular Mechanisms Linking Oxidative Stress to RVO Pathology

Collectively, oxidative stress amplifies retinal injury in RVO through endothelial dysfunction, coagulation abnormalities, activation of inflammatory and complement pathways, and dysregulation of cell death mechanisms. This mechanistic framework provides a strong theoretical rationale for combining antioxidant strategies with anti-VEGF therapy to achieve more comprehensive disease control in RVO.

## 5. Clinical and Experimental Evidence

Oxidative stress has been consistently demonstrated to play a central role in the pathogenesis of RVO. In this section, we summarize the available evidence from three complementary perspectives: (1) oxidative stress markers measured in clinical specimens, (2) associations between imaging findings and oxidative stress-related changes, and (3) the effects of oxidative stress-targeted interventions in animal models.

### 5.1. Oxidative Stress Markers in the Serum and Vitreous of Patients with RVO

Table 1 summarizes clinical studies reporting oxidative stress markers in patients with RVO. Clinical investigations have shown that patients with RVO exhibit significantly elevated serum levels of oxidative stress markers, including malondialdehyde (MDA), 8-hydroxy-2′-deoxyguanosine (8-OHdG), and hydrogen peroxide (H_2_O_2_), accompanied by reduced levels of antioxidant enzymes such as superoxide dismutase (SOD), catalase, and peroxisome proliferator-activated receptor gamma (PPAR-γ)-related molecules [15]. These findings suggest that localized retinal circulatory impairment may be closely associated with systemic oxidative burden.

More recent studies have further demonstrated that patients with RVO show increased levels of oxidative stress-related factors, including high-mobility group box 1 (HMGB1) and nitric oxide (NO), together with decreased antioxidant capacity, as reflected by reduced levels of GSH, SOD, and selenium. Importantly, these parameters have been proposed as potential prognostic biomarkers [36]. Collectively, these blood-based markers provide clinical evidence that disruption of the oxidative–antioxidative balance contributes to the progression and recurrence of RVO.

Although oxidative stress markers are not disease-specific, a growing body of clinical data supports a consistent association between retinal circulatory disorders and increased oxidative burden in RVO [7,14].

### 5.2. Associations Between Imaging Findings and Oxidative Stress-Related Changes

Table 2 summarizes associations between imaging findings (optical coherence tomography and fluorescein angiography) and oxidative stress-related factors in RVO. Optical coherence tomography (OCT) and fluorescein angiography (FA) are essential tools for quantitatively assessing retinal structural alterations and vascular abnormalities in RVO. Oxidative stress promotes the breakdown of the blood–retinal barrier (BRB) and increases vascular permeability, thereby exacerbating retinal edema and microhemorrhages.

Clinical imaging studies have suggested that increased inner retinal thickness and the severity of macular edema observed on OCT are associated with elevated oxidative stress markers in patients with RVO [14]. In FA, increased vascular leakage and expansion of microvascular non-perfusion areas are frequently observed, findings that are considered to reflect BRB disruption and inflammation- and oxidation-induced vascular injury.

In addition, noninvasive indicators of oxidative stress based on imaging modalities have recently attracted attention. Changes in endogenous fluorescent molecules, such as mitochondrial oxidative stress-related flavoprotein fluorescence, have been investigated across various retinal diseases. In RVO, increased flavoprotein fluorescence intensity has been reported in affected eyes, suggesting heightened mitochondrial oxidative stress [39]. These imaging-based findings may serve as novel quantitative indicators linking functional retinal alterations to underlying oxidative stress.

## 6. Antioxidant Therapeutic Strategies and Supporting Evidence

**Figure 5:**
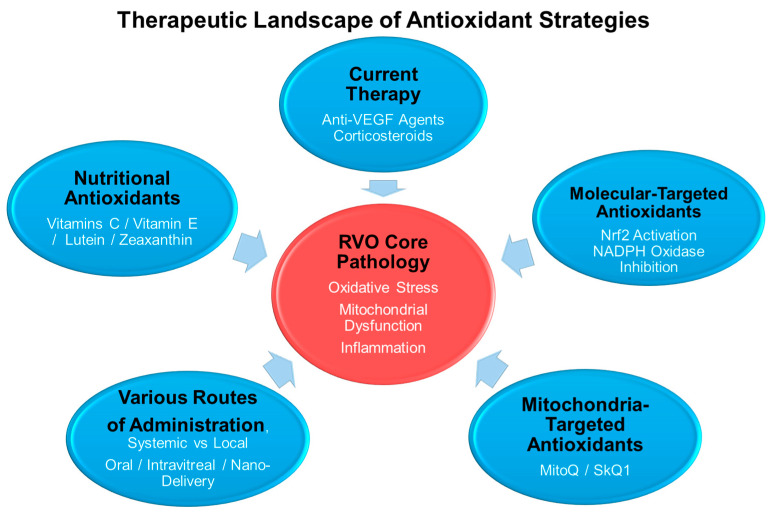
Therapeutic landscape of antioxidant strategies in retinal vein occlusion (RVO). Oxidative stress represents a central pathogenic mechanism in RVO, interacting with mitochondrial dysfunction, inflammation, and VEGF signaling. Current therapies such as anti-VEGF agents and corticosteroids primarily target vascular permeability and inflammation, whereas antioxidant strategies provide complementary approaches. These include nutritional antioxidants, molecular-targeted interventions (e.g., Nrf2 activation and NADPH oxidase inhibition), and mitochondria-targeted antioxidants. Various routes of administration, including systemic, local, and nano-delivery systems, are under investigation to optimize therapeutic efficacy and safety.

RVO is a pathological condition in which oxidative stress plays a central role, and antioxidant-based therapeutic strategies have therefore attracted considerable interest in both basic research and clinical applications [14]. This section summarizes the rationale for combining antioxidant therapy with current standard treatments, the types of antioxidants and their supporting evidence, and the current status and limitations of clinical trials. Figure 5 illustrates the therapeutic landscape and future perspectives of antioxidant strategies in RVO.

### 6.1. Integration with Current Clinical Approaches

#### 6.1.1. Positioning of Antioxidant Interventions in Combination with Anti-VEGF and Steroid Therapies

In patients with RVO complicated by macular edema, intravitreal anti-VEGF therapy has been widely established as the standard of care, demonstrating high efficacy in improving visual acuity and reducing macular edema [40]. However, anti-VEGF therapy typically provides short-term benefits, requires repeated injections, and may show limited efficacy in cases with persistent inflammation and oxidative stress [41].

Antioxidant interventions may therefore be positioned as complementary therapies that regulate oxidative stress independently of VEGF and inflammatory signaling pathways [7]. In addition to vascular permeability control achieved by anti-VEGF treatment, antioxidant therapies are expected to enhance BRB integrity and retinal neuronal survival by reducing ROS production and preserving mitochondrial function. The combination of anti-VEGF or steroid therapy with antioxidant approaches is theoretically rational, as it targets the mutually reinforcing loop between inflammation and oxidative stress [7].

#### 6.1.2. Management of Systemic Risk Factors and Antioxidant Nutrition

Systemic vascular risk factors for RVO, including hypertension, dyslipidemia, impaired glucose metabolism, and smoking, are all closely associated with chronic increases in oxidative stress [14]. Accordingly, management of these risk factors should form the foundation of any antioxidant therapeutic strategy.

Dietary interventions emphasizing antioxidant nutrition—such as increased intake of vegetables and fruits and supplementation with omega-3 polyunsaturated fatty acids (ω-3 PUFAs)—have been reported to attenuate oxidative and inflammatory states and to support overall retinal vascular health [38]. Reviews of dietary patterns associated with RVO have suggested that plant-based foods and anti-inflammatory nutrients may contribute to risk reduction [42]. In addition, oral supplementation with vitamin D has been suggested to improve macular edema in patients with central retinal vein occlusion, indicating that nutritional management may simultaneously address systemic risk factors and oxidative stress [42].

### 6.2. Types of Antioxidants and Supporting Evidence

The classification of antioxidant strategies presented in Table 3 is based on primary biological mechanisms of action rather than chemical structure alone. Specifically, agents were categorized according to whether they (1) directly scavenge reactive oxygen species, (2) selectively target mitochondrial ROS production, (3) activate endogenous antioxidant transcriptional pathways such as Nrf2, (4) inhibit ROS-generating enzymes including NADPH oxidases, (5) modulate glutathione homeostasis, or (6) employ advanced delivery systems to enhance retinal bioavailability. Representative agents were selected based on mechanistic evidence reported in MEDLINE-indexed experimental and translational studies.

Table 3 summarizes the major classes of antioxidants and their mechanisms of action. Antioxidant therapies can be broadly classified into several strategies based on their chemical and biological mechanisms. The principal categories and relevant evidence are outlined below.

#### 6.2.1. Nutritional Antioxidants: Vitamins and Carotenoids

Vitamin C and vitamin E are classical antioxidant molecules that directly scavenge ROS in vivo and are theoretically effective in reducing oxidative tissue damage. However, their clinical efficacy in retinal vascular diseases has not been conclusively established. In the Age-Related Eye Disease Study (AREDS), antioxidant and zinc supplementation was shown to slow the progression of age-related macular degeneration (AMD) (AREDS1/AREDS2), but this effect has not been validated in RVO [44].

Carotenoids such as lutein and zeaxanthin are naturally concentrated in the macula and have been suggested to exert antioxidative and photoprotective effects in the retina. These nutrients have been reported to correlate with healthier retinal vessel calibers, indicating a potential role in maintaining oxidative balance and vascular function [37]. Nevertheless, no randomized controlled trials specifically targeting patients with RVO have been reported to date.

#### 6.2.2. Mitochondria-Targeted Antioxidants and Intracellular ROS Regulation

Mitochondria are a major source of ROS, and mitochondria-targeted antioxidants, such as MitoQ, have been investigated as strategies to suppress intramitochondrial ROS accumulation. While preclinical studies in retinal diseases suggest potential benefits, clinical evidence specific to RVO remains limited. Antioxidant interventions in animal models have repeatedly been shown to attenuate ischemia–reperfusion injury, highlighting mitochondria-targeted antioxidant therapy as a promising future direction [7].

#### 6.2.3. NADPH Oxidase Inhibition and Activation of the Nrf2 Pathway

Activation of the Nrf2 pathway, a central transcriptional regulator of endogenous antioxidant responses, has attracted attention as a molecular target for suppressing oxidative stress. Nrf2 activation has demonstrated retinal protective effects in other retinal disease models, such as glaucoma, suggesting that similar mechanisms may be applicable to RVO pathophysiology [53]. Compounds such as sulforaphane and other Nrf2 activators have been shown in animal studies to induce antioxidant gene expression and enhance resistance to oxidative stress.

#### 6.2.4. Combined Antioxidant–Anti-Inflammatory Agents and Advanced Delivery Strategies

Recent research has focused on compounds with dual antioxidant and anti-inflammatory properties, as well as nanoparticle-based local delivery systems. For example, nanoparticle-mediated delivery of antioxidants has been reported to improve pharmacokinetics at target sites and to enhance BRB preservation and retinal protection [14]. Such combination therapies and high-efficiency delivery strategies represent promising future options for treating oxidative–inflammatory pathology in RVO.

### 6.3. Clinical Trials and Current Limitations

Given the translational relevance of oxidative stress modulation in RVO, this section integrates evidence from both preclinical animal studies and available clinical data. By consolidating experimental findings and clinical observations within a single framework, we aim to provide a comprehensive overview of the current therapeutic landscape, while clearly delineating existing limitations and future research priorities.

It is important to recognize that “antioxidant therapy” represents a heterogeneous category of interventions with distinct mechanisms of action. In this review, antioxidant strategies are classified into three major groups: (1) direct free radical scavengers that neutralizeROS, such as edaravone and hydrogen gas; (2) nutritional or polyphenolic antioxidants that exert combined antioxidant and anti-inflammatory effects but may be limited by systemic bioavailability; and (3) molecular modulators of endogenous antioxidant pathways, including activators of sirtuin 1 (SIRT1) or nuclear factor erythroid Nrf2, which enhance intrinsic cellular defense systems. Distinguishing these mechanistically distinct approaches is essential for understanding their translational potential and therapeutic limitations in RVO.

#### 6.3.1. Effects of Oxidative Stress-Targeted Interventions in Animal Models

Animal models of RVO, most commonly laser-induced retinal vein occlusion models, have been widely used to reproduce key pathological features such as vascular occlusion, reperfusion injury, and microcirculatory dysfunction. These models are well established as experimental platforms for evaluating the contribution of oxidative stress to RVO pathophysiology [54].

Several experimental studies have examined the effects of oxidative stress-targeted interventions using edaravone, a potent free radical scavenger. As summarized in the review by Masuda et al., edaravone was shown to suppress oxidative deoxyribonucleic acid (DNA) and lipid damage, reduce apoptosis, and attenuate neuronal and neurovascular injury in retinal ischemia–reperfusion models [7]. These protective effects were attributed, at least in part, to inhibition of the mitogen-activated protein kinase (MAPK) signaling pathway, indicating that antioxidant interventions may exert beneficial effects against dynamic, oxidative stress-driven pathological processes.

Furthermore, additional studies have reported that antioxidant treatment alleviates laser-induced pathological angiogenesis and retinal edema across multiple animal species [7,14]. Together, these experimental findings strongly support the concept that oxidative stress is a central driver of RVO pathogenesis and that antioxidant therapies hold promise as disease-modifying strategies.

Despite the robust protective effects observed in experimental models, important translational challenges remain. Differences in disease onset, vascular anatomy, inflammatory milieu, and systemic comorbidities between animal models and human RVO may influence therapeutic responsiveness. Therefore, careful consideration of timing, dosage, route of administration, and combination strategies with current standard therapies is essential before clinical implementation can be justified.

#### 6.3.2. Current Evidence and Gaps in Antioxidant Clinical Trials for RVO

To further clarify the current clinical research landscape, we conducted a systematic search of ClinicalTrials.gov (accessed 7 February 2026) using the terms “retinal vein occlusion”, “central retinal vein occlusion”, and “branch retinal vein occlusion” in combination with “antioxidant”, “oxidative stress”, “polyphenol”, “vitamin”, and “mitochondrial.” No registered interventional clinical trials specifically targeting oxidative stress in RVO were identified at the time of the search. This absence of registered trials underscores the substantial translational gap between robust preclinical evidence and clinical investigation in patients with RVO. It should also be considered that negative or inconclusive antioxidant trials may remain unpublished, potentially contributing to publication bias and limiting comprehensive assessment of therapeutic efficacy.

At present, large-scale randomized controlled trials specifically evaluating antioxidant therapies in patients with RVO are scarce. Although antioxidant nutrients and adjunctive therapies have been investigated in various ocular diseases, direct evidence from RVO-focused randomized trials remains limited. While antioxidant approaches have shown promise in other retinal diseases, such as AMD and diabetic retinopathy, robust clinical data for RVO are still lacking [55]. This gap partly reflects the difficulty of evaluating therapeutic interventions in the acute inflammatory and oxidative milieu characteristic of RVO.

In contrast, animal model studies have repeatedly demonstrated that modulation of oxidative stress can attenuate retinal ischemic injury and edema formation. Table 4 summarizes experimental studies of antioxidant interventions in RVO models. Tang et al. reported that treatment with the mitochondria-targeted antioxidant mitoquinone (MitoQ) improved retinal ischemia–reperfusion injury, suppressed ROS production, reduced cellular apoptosis, and improved retinal function in animal models [45]. The proposed mechanism involved regulation of SIRT1/Notch homolog 1 (Notch1)/NADPH oxidase signaling pathway. However, these findings remain limited to preclinical models, and no clinical trials in patients with RVO have yet been conducted.

Similarly, Hidaka et al. demonstrated that arctigenin, an orally available polyphenolic compound, significantly reduced retinal edema in a mouse model of RVO [56]. This effect was associated with preservation of tight junction-related proteins and downregulation of VEGF and tumor necrosis factor alpha (TNFα), suggesting concurrent antioxidant and anti-inflammatory actions. Nevertheless, clinical validation in human RVO has not been performed.

Edaravone, a clinically approved free radical scavenger, has been shown by Masuda et al. to suppress oxidative damage, apoptosis, and pathological angiogenesis in retinal disease models [7]. Although these findings are highly relevant to RVO pathophysiology, the studies encompassed multiple retinal disease models, and RVO-specific outcome assessments were limited.

Additionally, Long et al. reported that hydrogen gas inhalation therapy reduced retinal edema, shortened recanalization time after vascular occlusion, and improved retinal function in animal models [57]. These effects were suggested to be associated with reduced VEGF-α expression; however, the precise molecular mechanisms remain unclear, and clinical data in patients with RVO are currently unavailable.

From a clinical observational perspective, Altinisik et al. evaluated antioxidant indices in the aqueous humor and serum of patients with RVO, including total antioxidant status (TAS) and oxidative stress index (OSI), and reported that elevated oxidative stress levels were associated with greater severity of macular edema [13]. However, this study did not directly assess the therapeutic efficacy of antioxidant interventions.

Overall, these studies provide important preclinical and clinical indications that modulation of oxidative stress may contribute to disease modification in RVO. Nevertheless, most available evidence is derived from animal experiments, underscoring the need for prospective clinical trials to evaluate the efficacy, safety, and optimal treatment parameters of antioxidant therapies specifically in RVO.

Future research should focus on well-designed randomized controlled trials incorporating validated oxidative stress biomarkers to identify patient subgroups most likely to benefit from antioxidant strategies. Integration of molecular profiling, imaging biomarkers, and systemic risk assessment may enable personalized therapeutic approaches beyond VEGF-centered paradigms.

Another critical challenge in translating antioxidant strategies into clinical practice relates to pharmacokinetic and tissue delivery limitations. Many nutritional antioxidants and polyphenolic compounds exhibit low oral bioavailability due to limited intestinal absorption, extensive first-pass hepatic metabolism, and rapid systemic clearance. Furthermore, effective therapeutic action in RVO requires sufficient penetration across the blood–retinal barrier, adequate intracellular uptake, and, ideally, localization to mitochondria, where excessive reactive oxygen species (ROS) generation predominantly occurs during ischemia–reperfusion injury. Insufficient retinal and mitochondrial bioavailability may therefore partly explain the discrepancy between promising experimental findings and the limited clinical evidence observed to date. Advanced delivery strategies, including nanoparticle formulations and mitochondria-targeted antioxidants such as MitoQ, may help overcome these translational barriers.

#### 6.3.3. Challenges in Administration Route, Dosage, Treatment Duration, and Endpoint Selection

For antioxidant therapies to be clinically effective, appropriate administration routes (systemic versus local intravitreal delivery), dosage regimens, treatment duration, and clearly defined endpoints must be established. Moreover, because oxidative stress intersects with multiple metabolic and inflammatory pathways, evaluation of therapeutic efficacy using a single oxidative marker is often insufficient. These challenges in trial design represent critical considerations for the clinical implementation of antioxidant therapies in RVO.

## 7. Challenges in Clinical Translation and Future Research Directions

Unlike earlier reviews that primarily summarized antioxidant effects in general retinal pathology, this review emphasizes disease-specific mechanisms of oxidative injury in RVO, including venous stasis-induced ischemia, reperfusion-associated ROS burst, and VEGF-independent inflammatory pathways. By integrating mechanistic insights with clinical translational considerations, we aim to provide a more focused framework for future RVO-directed therapeutic development.

It is important to emphasize that the pathophysiology of RVO differs fundamentally from that of chronic retinal degenerative disorders such as AMD and diabetic retinopathy. RVO is primarily characterized by acute venous stasis, thrombotic occlusion, and ischemia–reperfusion injury, leading to a rapid surge in oxidative stress and inflammatory mediators. In contrast, AMD and diabetic retinopathy involve progressive, multifactorial degeneration driven by long-term metabolic, inflammatory, and microvascular alterations. Therefore, although oxidative stress represents a shared pathological component, the temporal dynamics and therapeutic targets may differ substantially between these conditions.

While substantial evidence supporting antioxidant strategies has been generated in AMD and diabetic retinopathy models, these findings should be interpreted cautiously when extrapolated to RVO. Evidence derived from other retinal diseases is presented in this review primarily to provide mechanistic insights into redox regulation and pathway modulation. Direct clinical evidence specific to RVO remains limited, underscoring the need for disease-focused investigations rather than reliance on cross-disease inference.

Table 5 summarizes key unresolved issues and proposed strategies for future research. Accumulating evidence indicates that oxidative stress plays a central role in the pathophysiology of RVO and that antioxidant strategies are theoretically promising therapeutic approaches [14]. Nevertheless, numerous challenges and unexplored areas remain before these strategies can be translated into routine clinical practice. This section outlines current limitations and future research directions from the perspectives of patient selection, biomarker-driven precision medicine, safety and drug–drug interactions, and translational models with long-term follow-up.

### 7.1. Optimization of Patient Selection and Treatment Timing (Central vs. Branch RVO; Acute vs. Chronic Phases)

RVO comprises two major clinical subtypes—CRVO and BRVO—which differ substantially in disease progression and therapeutic response [58]. CRVO is generally associated with a poorer prognosis, more severe acute inflammation and ischemia, and presumably a higher oxidative stress burden following disease onset. In contrast, BRVO typically represents a partial circulatory disturbance and often exhibits a more favorable clinical course with better visual recovery. Given these differences, it remains unclear whether the therapeutic efficacy of antioxidant interventions is consistent across RVO subtypes.

Similarly, the optimal timing of antioxidant intervention according to disease stage (acute versus chronic) has not been firmly established. Oxidative stress is generally elevated during the acute phase, when hemodynamic disturbance, ischemia, and inflammation are most pronounced, suggesting that antioxidant therapy may exert greater benefits during this period. However, persistent oxidative–inflammatory loops may also operate in the chronic phase, indicating that intervention at later stages may still hold therapeutic value [14].

Accordingly, defining patient selection criteria based on disease subtype and onset timing, as well as quantitatively determining the therapeutic window for oxidative stress-targeted interventions, represents a major challenge in clinical trial design. In particular, studies comparing treatment responses between CRVO and BRVO, as well as early versus delayed intervention and their impact on long-term outcomes, are urgently needed.

### 7.2. Biomarker-Based Individualized Therapy (Precision Medicine)

Given the clinical relevance of oxidative stress in RVO, the development of biomarker-driven individualized treatment strategies has emerged as an important research direction. Recent studies have suggested that oxidative–antioxidative factors in serum and vitreous fluid—such as selenium (Se), reduced GSH, superoxide dismutase (SOD), and high-mobility group box 1 (HMGB1)—may contribute to the diagnosis and prognostic prediction of RVO [36]. These quantitative biomarkers may reflect disease-specific oxidative burden and therapeutic responsiveness.

In addition, imaging biomarkers derived from OCT and OCTA are widely used to visualize blood flow alterations and microstructural changes in the retina. Integrating these imaging parameters with oxidative stress-related biomarkers may enable more precise prediction of visual outcomes and responses to edema resolution [59]. Such multidimensional biomarker-based precision medicine approaches have the potential to “target” antioxidant interventions and to individualize optimal treatment strategies even among patients with the same RVO diagnosis.

However, biomarker research in this field remains in an early stage. The standardization of measurement methods, validation of clinical predictive power, and harmonization across studies are essential. Furthermore, integrated analyses examining correlations between systemic biomarkers, local retinal environments (vitreous fluid), and OCT-based indices represent important future research priorities.

### 7.3. Safety and Drug–Drug Interaction Assessment of Antioxidant Therapies

The clinical implementation of antioxidant therapies requires rigorous evaluation of safety and potential drug–drug interactions. Combination therapy with anti-VEGF agents or steroids is common in clinical practice; however, data regarding interactions between these established treatments and antioxidant interventions remain limited. Although anti-VEGF therapy is generally considered safe even with long-term administration, the effects of specific antioxidants on vascular permeability, intracellular signaling pathways, or endogenous retinal repair mechanisms have not been thoroughly investigated [60].

In addition, antioxidants may exert off-target effects, and systemic alterations in redox balance could influence the function of other organs or the pathophysiology of comorbid conditions, such as cardiovascular disease or diabetes mellitus. This issue is particularly relevant in elderly patients and those with multiple comorbidities. Future clinical studies should therefore incorporate comprehensive assessments of safety profiles, pharmacological interactions, and long-term systemic health risks.

### 7.4. Translational Models and Long-Term Follow-Up Studies Bridging Bench and Bedside

Finally, successful clinical translation of antioxidant therapies requires robust translational research that bridges animal models and human studies. Currently, retinal ischemia–reperfusion models and laser-induced RVO models are widely used to investigate disease mechanisms and therapeutic effects. However, the extent to which these models faithfully recapitulate human RVO pathophysiology and oxidative stress dynamics remains insufficiently validated [7].

Moreover, long-term follow-up studies are needed to evaluate the impact of antioxidant interventions on visual prognosis, disease recurrence, and the development of complications such as retinal neovascularization. While most clinical trials focus on short-term improvements in visual function, the benefits of oxidative stress modulation may manifest as long-term tissue protection and delayed disease progression.

A critical translational challenge lies in the temporal dynamics of oxidative stress during ischemia–reperfusion injury. Experimental studies demonstrate that ROS generation during reperfusion occurs as a rapid and transient burst, largely originating from mitochondrial dysfunction and NADPH oxidase activation. While direct free radical scavengers are theoretically capable of neutralizing ROS, their clinical efficacy depends on achieving sufficient intra-retinal and intracellular concentrations precisely during this narrow therapeutic window. In practice, systemic administration may fail to deliver adequate drug levels to retinal mitochondria at the critical time point, thereby limiting clinical effectiveness despite promising preclinical results.

In animal models, antioxidant agents are often administered prophylactically or immediately at the onset of experimentally induced ischemia, conditions that are rarely replicated in clinical settings where treatment is initiated after symptom onset. Moreover, experimental models typically involve controlled and homogeneous injury patterns, whereas human RVO is influenced by systemic comorbidities, vascular aging, and heterogeneous inflammatory responses. These discrepancies may partly explain why direct scavengers demonstrate robust protective effects in preclinical ischemia–reperfusion models but yield limited or inconsistent benefits in human trials.

These limitations have prompted increasing interest in strategies that modulate endogenous antioxidant defense systems rather than relying solely on direct ROS neutralization. Activation of transcriptional regulators such as Nrf2 enhances the expression of multiple cytoprotective enzymes, including superoxide dismutase, catalase, and heme oxygenase-1, thereby providing sustained redox homeostasis. Unlike direct scavengers, pathway modulators may confer broader and longer-lasting cellular resilience against oxidative injury, potentially overcoming the narrow therapeutic window associated with acute ROS bursts.

Therefore, establishing an integrated research framework that combines improved preclinical models, identification of translational biomarkers, and long-term observational and interventional studies will be critical for the successful clinical adoption of antioxidant therapies in RVO.

### 7.5. Future Research Directions and Their Potential Impact on Visual Outcomes in RVO

Future research should focus on several key areas. First, the development and standardization of multidimensional biomarkers will be critical for risk stratification and implementation of individualized treatment strategies, consistent with the principles of precision medicine. Early identification of patients with high oxidative stress burden may enable targeted intervention and ultimately improve visual outcomes.

Second, the clinical evaluation of antioxidant interventions as part of integrated treatment strategies is required. In particular, whether antioxidants can exert synergistic effects when combined with anti-VEGF or steroid therapies, contribute to sustained resolution of macular edema, or reduce recurrence rates should be clarified through long-term follow-up studies.

Third, ongoing exploration of novel antioxidant agents and pathway-targeted therapies—such as Nrf2 activators and mitochondria-targeted compounds—continues to expand therapeutic possibilities. These approaches, which differ mechanistically from conventional treatments, aim to protect the entire retinal neurovascular unit and may represent a new class of disease-modifying therapies for RVO [14].

Ultimately, deeper understanding of oxidative stress biology and successful clinical translation of antioxidant strategies may not only improve visual prognosis for patients with RVO but also contribute to a broader shift in preventive and therapeutic paradigms for retinal vascular diseases as a whole.

## 8. Summary of the Role of Oxidative Stress in RVO

**Figure 6:**
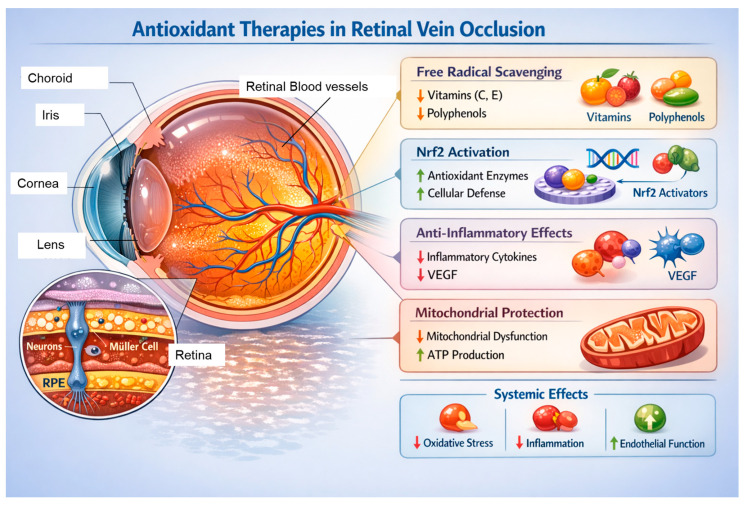
Summary of Antioxidant Strategies and Their Pathophysiological Targets in RVO. This figure illustrates the interplay between oxidative stress-induced damage and the potential therapeutic targets of various antioxidant interventions in the context of RVO. Anatomical Targets and Molecular Mechanisms: In the retina, excessive ROS generation leads to endothelial dysfunction, disruption of the BRB, and damage to the RPE and neurosensory retina (including ganglion cells and glial cells). Antioxidant Interventions: Polyphenols (e.g., Resveratrol, Curcumin): Act through ROS scavenging and the inhibition of pro-inflammatory pathways such as NF-κB. Vitamins (e.g., Vitamin C and E): Provide direct antioxidant effects by neutralizing free radicals and preventing lipid peroxidation in the vitreous and retinal tissues. Nrf2 Pathway Activators: Enhance the endogenous antioxidant defense system by upregulating cytoprotective enzymes (e.g., HO-1, NQO1) to mitigate ischemic injury. Clinical Potential: The diagram underscores the importance of the timing of intervention (acute vs. chronic phases) to improve long-term visual outcomes and reduce macular edema.

RVO is a retinal vascular disorder that can severely compromise visual prognosis and has traditionally been interpreted primarily in terms of inflammation and hemodynamic disturbances. However, accumulating evidence now indicates that oxidative stress plays a central role in the onset, progression, and visual dysfunction associated with RVO [14]. This section summarizes the role of oxidative stress in RVO, discusses the translational potential and practical challenges of antioxidant strategies, and outlines how future research may contribute to improved visual outcomes. An overview of the role of oxidative stress in RVO is presented in Figure 6.

### 8.1. Summary of the Role of Oxidative Stress in RVO and Its Clinical Implications

Multiple studies have demonstrated significant alterations in oxidative stress markers in patients with RVO, including changes in serum and aqueous humor levels of MDA, 8-OHdG, and global indices of oxidative and antioxidative capacity, which correlate with disease activity and prognosis [15]. In addition, oxidative stress affects blood components and vascular endothelial function, exacerbating microcirculatory impairment through reduced erythrocyte membrane deformability and altered blood viscosity [16]. Collectively, these findings suggest that redox imbalance is deeply involved in RVO pathophysiology and may function not merely as a downstream consequence but as a key driver of disease progression.

These observations carry important clinical implications. Assessment of oxidative stress may provide valuable information for evaluating disease severity and predicting therapeutic response in patients with RVO, supporting its potential utility as a diagnostic and prognostic biomarker. For example, combinations of redox-related factors such as serum selenium, GSH, and SOD have been reported to improve diagnostic accuracy and prognostic prediction in RVO [36]. Such molecular-level indicators may complement conventional clinical parameters, including visual acuity, OCT findings, and fluorescein angiography, by capturing aspects of disease biology that are otherwise difficult to assess.

### 8.2. Translational Potential and Practical Challenges of Antioxidant Strategies

A wide range of antioxidant strategies has been proposed, including free radical scavengers (e.g., edaravone), nutritional antioxidants (vitamins C and E, lutein, and zeaxanthin), mitochondria-targeted antioxidants, and activation of endogenous protective pathways such as Nrf2 and SIRT1. Despite their biological plausibility, large-scale clinical trials demonstrating clear efficacy of these approaches in RVO are still lacking [7].

In animal models, antioxidant agents have been shown to suppress oxidative damage to DNA and lipids and to confer structural and functional protection to retinal tissue, reducing both neuronal and vascular injury [7]. These findings support the biological rationale for targeting oxidative stress; however, substantial challenges remain in translating these results from bench to bedside.

Key issues include (i) identification and specificity of therapeutic targets, (ii) selection of appropriate patient populations (acute vs. chronic disease, CRVO vs. BRVO), (iii) optimization of administration routes, dosage, and treatment duration, and (iv) establishment of effective and standardized clinical endpoints to assess treatment response. In addition, antioxidant therapy is unlikely to be used in isolation; integration with anti-VEGF and anti-inflammatory treatments, as well as coordination with systemic risk factor management, will be essential.

Although antioxidant therapies are generally regarded as having favorable safety profiles, data regarding off-target effects, long-term administration, and drug–drug interactions remain limited. Therefore, future clinical trial designs must incorporate comprehensive safety evaluations alongside efficacy assessments.

## 9. Conclusions (Final Remarks)

RVO is increasingly recognized as a retinal vascular disorder in which oxidative stress plays a central and active role in disease initiation, progression, and visual impairment. Clinical and experimental evidence demonstrates that redox imbalance contributes to endothelial dysfunction, microcirculatory disturbance, neuronal injury, and inflammatory amplification, positioning oxidative stress not merely as a secondary consequence but as a key pathogenic driver. Although multiple antioxidant strategies—including free radical scavengers, nutritional antioxidants, mitochondria-targeted agents, and endogenous pathway activators—have shown promising protective effects in experimental models, robust clinical evidence in RVO remains limited. Future studies should focus on target specificity, patient stratification, optimized therapeutic protocols, standardized clinical endpoints, and integration with established treatments such as anti-VEGF therapy. A deeper understanding of redox mechanisms and carefully designed translational trials will be essential to determine whether antioxidant strategies can meaningfully improve visual outcomes in patients with RVO.

## Figures and Tables

**Table 1:** Clinical studies evaluating oxidative stress markers in patients with RVO.

Author	Year	RVO Type	Study Population	Sample	Oxidative Stress Markers	Key Findings
Chen et al. [15]	2019	CRVO/BRVO	RVO patients vs. healthy controls	Serum	MDA, 8-OHdG, H_2_O_2_, SOD, Catalase	Significantly higher levels of MDA, 8-OHdG, and H_2_O_2_, with reduced SOD and catalase activity in RVO patients, indicating increased systemic oxidative stress
Masuda et al. [7]	2024	BRVO	RVO patients	Vitreous fluid	MDA, 8-OHdG	Vitreous levels of lipid peroxidation and DNA oxidation markers were significantly elevated, supporting the role of local retinal oxidative stress
Shen et al. [36]	2025	CRVO/BRVO	RVO patients	Serum	HMGB1, NO, GSH, SOD, Selenium	Increased oxidative stress-related factors (HMGB1, NO) and decreased antioxidant capacity (GSH, SOD, selenium); several markers were associated with visual prognosis
Zhang et al. [14]	2025	Mixed RVO	RVO patients	Serum	MDA, TAC, SOD	Elevated oxidative stress markers and reduced total antioxidant capacity (TAC) correlated with disease severity
Kumari et al. [37]	2018	Retinal vascular disease	Patients with retinal vascular disorders	Serum	Carotenoids, Oxidative index	Lower carotenoid levels were associated with increased oxidative burden and retinal vascular dysfunction
Connor et al. [38]	2007	Retinal circulation-related risk	High-risk population	Serum	PUFA, Oxidative markers	Reduced antioxidant nutrient status was associated with increased oxidative stress and impaired retinal vascular health

Abbreviations: RVO, retinal vein occlusion; CRVO, central retinal vein occlusion; BRVO, branch retinal vein occlusion; MDA, malondialdehyde; 8-OHdG, 8-hydroxy-2′-deoxyguanosine; SOD, superoxide dismutase; TAC, total antioxidant capacity; HMGB1, high mobility group box 1; NO, nitric oxide; GSH, glutathione; PUFA, polyunsaturated fatty acids.

**Table 2:** Associations between Imaging Findings (OCT/FA) and Oxidative Stress-Related Factors in RVO.

Imaging Finding (OCT/FA)	Oxidative Stress-Related Factors	Pathophysiological Significance
Retinal edema/Macular edema	Reactive oxygen species (ROS), VEGF	Breakdown of the blood–retinal barrier (BRB) and increased vascular permeability
Increased inner retinal thickness (OCT)	Oxidative stress markers	Structural retinal changes associated with oxidative injury and inflammation
Microaneurysms/Microhemorrhages (FA)	ROS, Inflammatory mediators	Oxidative stress-induced microvascular damage
Capillary non-perfusion areas (FA)	Hypoxia, Oxidative stress	Ischemia–reperfusion injury and progression of retinal ischemia
Increased vascular leakage (FA)	VEGF, ROS	BRB disruption and oxidative stress-mediated endothelial dysfunction
Increased flavoprotein fluorescence	Mitochondrial oxidative stress	Indicator of mitochondrial dysfunction and oxidative stress burden

Abbreviations: OCT, optical coherence tomography; FA, fluorescein angiography; ROS, reactive oxygen species; VEGF, vascular endothelial growth factor; BRB, blood–retinal barrier; RVO, retinal vein occlusion. Notes: Imaging findings obtained by OCT and FA reflect structural and microvascular alterations associated with oxidative stress-related pathways in RVO. The associations summarized in this table are based on previously published clinical and experimental studies and do not necessarily indicate direct causality. Non-invasive imaging biomarkers, including flavoprotein fluorescence, are considered surrogate indicators of mitochondrial oxidative stress.

**Table 3:** Mechanism-based classification of antioxidant strategies targeting oxidative stress pathways in RVO.

Category	Representative Agents	Primary Targets/Mechanisms	Level of Evidence	Key References
Nutritional antioxidants	Vitamin	Direct scavenging of ROS; Reduction in lipid peroxidation	Observational studies	Lendzioszek 2023 [42]; Daneshvar 2024 [43]
Nutritional antioxidants	Lutein, Zeaxanthin	Macular antioxidant activity; Blue light filtering; Vascular protection	Observational studies	AREDS 2001 [44]
Mitochondria-targeted antioxidants	MitoQ	Reduction in mitochondrial ROS; Protection against ischemia–reperfusion injury	Animal studies	Tang 2022 [45]
Mitochondria-targeted antioxidants	SkQ1	Mitochondrial membrane stabilization; ROS suppression	Experimental/Animal studies	Perepechaeva 2014 [46]
Nrf2 pathway activators	Sulforaphane	Activation of Nrf2-dependent antioxidant genes (HO-1, NQO1)	Experimental/Animal studies	Pan 2014 [47]
Nrf2 pathway activators	Bardoxolone methyl	Enhancement of endogenous antioxidant responses via Nrf2	Experimental studies	Chien 2021 [48]
NADPH oxidase inhibitors	Apocynin	Inhibition of ROS production via NADPH oxidase	Animal studies	Saito 2007 [49]
GSH-related agents	N-acetylcysteine (NAC)	GSH replenishment; Intracellular redox balance	Experimental/Limited clinical data	Wood 2024 [50]
Combined antioxidant–anti-inflammatory agents	Polyphenols (e.g., resveratrol)	ROS reduction; Inhibition of inflammatory signaling	Animal studies	Chronopoulos 2023 [51]
Advanced delivery strategies	Nanoparticle-based antioxidants	Targeted retinal delivery; BRB protection	Preclinical studies	Shahror 2025 [52]

Abbreviations: RVO, retinal vein occlusion; SkQ1, 10-(6′-plastoquinonyl) decyltriphenylphosphonium; ROS, reactive oxygen species; AREDS, Age-Related Eye Disease Study; MitoQ, mitoquinone; Nrf2, nuclear factor erythroid 2-related factor 2; HO-1, heme oxygenase-1; NQO1, NAD(P)H quinone dehydrogenase 1; NADPH, nicotinamide adenine dinucleotide phosphate; GSH, glutathione; NAC, N-acetylcysteine; BRB, blood–retinal barrier.

**Table 4:** Evidence-based summary of preclinical and clinical antioxidant intervention studies in RVO.

Model	Intervention	Outcome	Limitations
Animal-MitoQ [45]	MitoQ	Improved retinal ischemia–reperfusion injury, reduced ROS, suppressed apoptosis, improved retinal function	Preclinical; not yet tested in RVO patients; Mechanism via SIRT1/Notch1/NADPH axis
Animal-Arctigenin [56]	Arctigenin (oral polyphenol)	Reduced retinal edema in RVO mouse model by preserving junction proteins, lowered VEGF and TNFα expression	Preclinical; antioxidant + anti-inflammatory effects; Clinical translation untested
Animal-Edaravone [7]	Edaravone (free radical scavenger)	Decreased oxidative damage, apoptosis, and angiogenesis in retinal disease models including RVO	Evidence mostly from combined retinal disease models; Specific RVO outcome measures limited
Animal-Hydrogen gas inhalation [57]	Hydrogen gas	Alleviated retinal edema, shortened occlusion reopening, improved retinal function; possibly via decreased VEGF-α	Preclinical; mechanism not fully defined; Human data lacking
Clinical-Antioxidant indices (aqueous humor/serum) [13]	Natural antioxidant status (TAS, OSI)	Higher oxidative stress associated with macular edema severity in RVO patients	Observational; Not an interventional study

Model definitions: Animal: Preclinical studies using rodent models of RVO and ischemia–reperfusion retinal injury. Clinical: Biomarker studies in human RVO patients (non-interventional). Abbreviations: RVO, retinal vein occlusion; ROS, reactive oxygen species; MitoQ, mitoquinone (mitochondria-targeted antioxidant); SIRT1, sirtuin 1; NADPH, nicotinamide adenine dinucleotide phosphate; VEGF, vascular endothelial growth factor; TNFα, tumor necrosis factor-alpha; TAS, total antioxidant status; OSI, oxidative stress index.

**Table 5:** Future challenges and potential strategies for antioxidant-based therapy in retinal vein occlusion.

Challenge	Key Issues	Potential Strategies
Patient selection	Heterogeneity between CRVO and BRVO, and between acute and chronic disease stages	Stratification based on RVO subtype and disease stage; Subtype-specific and stage-specific analyses in clinical trials
Optimal treatment timing	Uncertainty regarding the therapeutic window for antioxidant intervention	Prospective studies comparing early versus delayed intervention; Definition of the optimal therapeutic window
Lack of standardized biomarkers	Inconsistent measurement methods and cut-off values for oxidative stress markers	Standardization of circulating and intraocular oxidative stress biomarkers (e.g., GSH, SOD, HMGB1)
Limited precision medicine approaches	Large inter-individual variability in oxidative stress burden and treatment response	Integration of molecular biomarkers with imaging parameters (OCT/OCTA) to enable personalized therapy
Safety and drug–drug interactions	Insufficient data on interactions with anti-VEGF agents and corticosteroids	Dedicated combination studies assessing safety, pharmacodynamics, and interactions with standard therapies
Insufficient long-term safety data	Unknown systemic effects, Especially in elderly patients with comorbidities	Long-term follow-up studies evaluating systemic safety and visual outcomes
Limitations of current animal models	Incomplete recapitulation of human RVO pathology and oxidative stress dynamics	Development and validation of refined animal models that better mimic human RVO
Translational gap between preclinical and clinical studies	Difficulty translating preclinical antioxidant efficacy into clinical benefit	Identification of translational biomarkers linking preclinical outcomes to clinical endpoints
Endpoint selection in clinical trials	Overreliance on short-term visual acuity outcomes	Inclusion of long-term endpoints such as retinal integrity, recurrence rates, and neovascular complications

Abbreviations: RVO, retinal vein occlusion; CRVO: central retinal vein occlusion, BRVO, branch retinal vein occlusion; OCT, optical coherence tomography; OCTA, optical coherence tomography angiography; GSH, glutathione; SOD, superoxide dismutase; HMGB1, high-mobility group box 1 protein; anti-VEGF, anti-vascular endothelial growth factor.

## Data Availability

No new data were created or analyzed in this study. Data sharing is not applicable to this article.

## Abbreviations

The following abbreviations are used in this manuscript:

AMD	Age-related macular degeneration
AP-1	Activator protein-1
AREDS	Age-Related Eye Disease Study
BRB	blood–retinal barrier
BRVO	Branch retinal vein occlusion
CRVO	Central retinal vein occlusion
DHA	Docosahexaenoic acid
DNA	Deoxyribonucleic acid
GSH	Glutathione
HMGB1	High mobility group box 1
IL-6	Interleukin-6
MAC	Membrane attack complex
MAPK	Mitogen-activated protein kinase
MCP-1	Monocyte chemoattractant protein-1
MDA	Malondialdehyde
MitoQ	Mitoquinone (mitochondria-targeted antioxidant)
MPO	Myeloperoxidase
NADPH	Nicotinamide adenine dinucleotide phosphate
NETs	Neutrophil extracellular traps
NF-κB	Nuclear factor kappa-light-chain-enhancer of activated B cells
NHANES	National Health and Nutrition Examination Survey
NO	Nitric oxide
NOX	NADPH oxidase
Nrf2	Nuclear factor erythroid 2-related factor 2
LDL	Low-density lipoprotein
OCT	Optical coherence tomography
OSI	Oxidative stress index
PUFA(s)	Polyunsaturated fatty acid(s)
RPE	Retinal pigment epithelium
ROS	Reactive oxygen species
RVO	Retinal vein occlusion
SIRT1	Sirtuin 1
SkQ1	10-(6′-plastoquinonyl) decyltriphenylphosphonium
SOD	Superoxide dismutase
TAC	Total antioxidant capacity
TAS	Total antioxidant status
TNF-α	Tumor necrosis factor alpha
VEGF	Vascular endothelial growth factor
ZO-1	Zonula occludens-1
4-HNE	4-hydroxynonenal
8-OHdG	8-hydroxy-2′-deoxyguanosine

## References

[1] Rogers S., McIntosh R.L., Cheung N., Lim L., Wang J.J., Mitchell P., Kowalski J.W., Nguyen H., Wong T.Y. (2010). International Eye Disease Consortium. The prevalence of retinal vein occlusion: Pooled data from population studies from the United States, Europe, Asia, and Australia. Ophthalmology.

[2] Hayreh S.S. (1983). Classification of central retinal vein occlusion. Ophthalmology.

[3] Rehak M., Wiedemann P. (2010). Retinal vein thrombosis: Pathogenesis and management. J. Thromb. Haemost..

[4] Beatty S., Koh H., Henson D., Boulton M. (2000). The role of oxidative stress in the pathogenesis of age-related macular degeneration. Surv. Ophthalmol..

[5] Campochiaro P.A., Bhisitkul R.B., Shapiro H., Rubio R.G. (2013). Vascular endothelial growth factor promotes progressive retinal nonperfusion in patients with retinal vein occlusion. Ophthalmology.

[6] Noma H., Mimura T., Shimada K. (2014). Role of inflammation in previously untreated macular edema with branch retinal vein occlusion. BMC Ophthalmol..

[7] Masuda T., Shimazawa M., Hara H. (2017). Retinal diseases associated with oxidative stress and the effects of a free radical scavenger (edaravone). Oxidative Med. Cell. Longev..

[8] Song P., Xu Y., Zha M., Zhang Y., Rudan I. (2019). Global epidemiology of retinal vein occlusion: A systematic review and meta-analysis of prevalence, incidence, and risk factors. J. Glob. Health.

[9] Kalva P., Akram R., Zuberi H.Z., Kooner K.S. (2023). Prevalence and risk factors of retinal vein occlusion in the United States: The National Health and Nutrition Examination Survey, 2005–2008. Bayl. Univ. Med. Cent. Proc..

[10] Jaulim A., Ahmed B., Khanam T., Chatziralli I.P. (2013). Branch retinal vein occlusion: Epidemiology, pathogenesis, risk factors, clinical features, diagnosis, and complications. Retina.

[11] Valeriani E., Paciullo F., Porfidia A., Pignatelli P., Candeloro M., Di Nisio M., Donadini M.P., Mastroianni C.M., Pola R., Gresele P. (2023). Antithrombotic treatment for retinal vein occlusion: A systematic review and meta-analysis. J. Thromb. Haemost..

[12] Noma H., Yasuda K., Nonaka R., Sasaki S., Shimura M. (2023). Anti-vascular endothelial growth factor therapy with or without initial steroid therapy for macular edema in branch retinal vein occlusion. Clin. Ophthalmol..

[13] Altinisik M., Koytak A., Elbay A., Toklu E., Sezer T., Kocyigit A. (2018). Oxidant–antioxidant balance in the aqueous humor of patients with retinal vein occlusion. Semin. Ophthalmol..

[14] Zhang J., Xie X., Mo Y. (2025). Exploring the role of oxidative stress in retinal vein occlusion: An updated and comprehensive review on the pathophysiology and treatment perspectives. Int. Ophthalmol..

[15] Chen K.H., Hsiang E.L., Hsu M.Y., Chou Y.C., Lin T.C., Chang Y.L., Tsai C.Y., Li T.H., Woung L.C., Chen S.J. (2019). Elevation of serum oxidative stress in patients with retinal vein occlusions. Acta Ophthalmol..

[16] Becatti M., Marcucci R., Gori A.M., Mannini L., Grifoni E., Alessandrello Liotta A., Sodi A., Tartaro R., Taddei N., Rizzo S. (2016). Erythrocyte oxidative stress is associated with cell deformability in patients with retinal vein occlusion. J. Thromb. Haemost..

[17] Wang Q., Zennadi R. (2020). Oxidative stress and thrombosis during aging: The roles of oxidative stress in RBCs in venous thrombosis. Int. J. Mol. Sci..

[18] Liu Y., Zhang D., Wu Y., Ji B. (2014). Docosahexaenoic acid aggravates photooxidative damage in retinal pigment epithelial cells via lipid peroxidation. J. Photochem. Photobiol. B.

[19] Cheng Y.S., Linetsky M., Gu X., Ayyash N., Gardella A., Salomon R.G. (2019). Light-induced generation and toxicity of docosahexaenoate-derived oxidation products in retinal pigmented epithelial cells. Exp. Eye Res..

[20] Hu L., Guo J., Zhou L., Zhu S., Wang C., Liu J., Hu S., Yang M., Lin C. (2020). Hydrogen sulfide protects retinal pigment epithelial cells from oxidative stress-induced apoptosis and affects autophagy. Oxid. Med. Cell. Longev..

[21] Granger D.N., Kvietys P.R. (2015). Reperfusion injury and reactive oxygen species: The evolution of a concept. Redox Biol..

[22] Wang J., Li M., Geng Z., Khattak S., Ji X., Wu D., Dang Y. (2022). Role of oxidative stress in retinal disease and the early intervention strategies: A review. Oxid. Med. Cell. Longev..

[23] Lessiani G., Vazzana N., Cuccurullo C., Di Michele D., Laurora G., Sgrò G., Di Ruscio P., Simeone E., Di Iorio P., Lattanzio S. (2011). Inflammation, oxidative stress and platelet activation in aspirin-treated critical limb ischaemia: Beneficial effects of iloprost. Thromb. Haemost..

[24] Fuchs T.A., Brill A., Duerschmied D., Schatzberg D., Monestier M., Myers D.D., Wrobleski S.K., Wakefield T.W., Hartwig J.H., Wagner D.D. (2010). Extracellular DNA traps promote thrombosis. Proc. Natl. Acad. Sci. USA.

[25] Devi V.L.J., Panigrahi P.K., Minj A., Hanisha D. (2025). Clinical profile of patients with retinal vein occlusion and its correlation with serum homocysteine levels. BMC Ophthalmol..

[26] Hankey G.J., Eikelboom J.W. (1999). Homocysteine and vascular disease. Lancet.

[27] Jiang H., Zhou Y., Nabavi S.M., Sahebkar A., Little P.J., Xu S., Weng J., Ge J. (2022). Mechanisms of oxidized LDL-mediated endothelial dysfunction and its consequences for the development of atherosclerosis. Front. Cardiovasc. Med..

[28] Jarrett S.G., Lin H., Godley B.F., Boulton M.E. (2008). Mitochondrial DNA damage and its potential role in retinal degeneration. Prog. Retin. Eye Res..

[29] Bedard K., Krause K.H. (2007). The NOX family of ROS-generating NADPH oxidases: Physiology and pathophysiology. Physiol. Rev..

[30] Ungvari Z., Tarantini S., Donato A.J., Galvan V., Csiszar A. (2018). Mechanisms of vascular aging. Circ. Res..

[31] Antonetti D.A., Barber A.J., Bronson S.K., Freeman W.M., Gardner T.W., Jefferson L.S., Kester M., Kimball S.R., Krady J.K., LaNoue K.F. (2006). JDRF Diabetic Retinopathy Center Group. Diabetic retinopathy: Seeing beyond glucose-induced microvascular disease. Diabetes.

[32] Ayala A., Muñoz M.F., Argüelles S. (2014). Lipid peroxidation: Production, metabolism, and signaling mechanisms of malondialdehyde and 4-hydroxy-2-nonenal. Oxid. Med. Cell. Longev..

[33] Zipfel P.F., Skerka C. (2009). Complement regulators and inhibitory proteins. Nat. Rev. Immunol..

[34] Redza-Dutordoir M., Averill-Bates D.A. (2016). Activation of apoptosis signalling pathways by reactive oxygen species. Biochim. Biophys. Acta Mol. Cell Res..

[35] Markitantova Y., Simirskii V. (2025). Retinal pigment epithelium under oxidative stress: Chaperoning autophagy and beyond. Int. J. Mol. Sci..

[36] Shen D., Li M., Sun W., Tao Z. (2025). Diagnostic and prognostic predictive value of serum selenium and redox biomarkers in retinal vein occlusion. Int. J. Gen. Med..

[37] Kumari N., Cher J., Chua E., Hamzah H., Wong T.Y., Cheung C.Y. (2018). Association of serum lutein and zeaxanthin with quantitative measures of retinal vascular parameters. PLoS ONE.

[38] Connor K.M., SanGiovanni J.P., Lofqvist C., Aderman C.M., Chen J., Higuchi A., Hong S., Pravda E.A., Majchrzak S., Carper D. (2007). Increased dietary intake of omega-3 polyunsaturated fatty acids reduces pathological retinal angiogenesis. Nat. Med..

[39] Ahsanuddin S., Rios H.A., Otero-Marquez O., Macanian J., Zhou D., Rich C., Rosen R.B. (2023). Flavoprotein fluorescence elevation is a marker of mitochondrial oxidative stress in patients with retinal disease. Front. Ophthalmol..

[40] Garay-Aramburu G., Hunt A., Arruabarrena C., Mehta H., Invernizzi A., Gabrielle P.H., Guillaumie T., Wolff B., Gillies M.C., Zarranz-Ventura J. (2024). Initial response and 12-month outcomes after commencing dexamethasone or vascular endothelial growth factor inhibitors for retinal vein occlusion in the FRB registry. Sci. Rep..

[41] Xu X., Han N., Zhao F., Fan R., Guo Q., Han X., Liu Y., Luo G. (2024). Inefficacy of anti-VEGF therapy reflected in VEGF-mediated photoreceptor degeneration. Mol. Ther. Nucleic Acids.

[42] Lendzioszek M., Mrugacz M., Bryl A., Poppe E., Zorena K. (2023). Prevention and treatment of retinal vein occlusion: The role of diet—A review. Nutrients.

[43] Daneshvar K., Akhlaghi M., Iranpour S., Irajpour M., Pourazizi M. (2024). Vitamin D deficiency in patients with retinal vein occlusion: A systematic review and meta-analysis. Int. J. Retin. Vitr..

[44] Age-Related Eye Disease Study Research Group (2001). A randomized, placebo-controlled, clinical trial of high-dose supplementation with vitamins C and E, beta carotene, and zinc for age-related macular degeneration and vision loss: AREDS report no. 8. Arch. Ophthalmol..

[45] Tang D., Liu X., Chen J. (2022). Mitoquinone intravitreal injection ameliorates retinal ischemia–reperfusion injury in rats involving the SIRT1/Notch1/NADPH axis. Drug Dev. Res..

[46] Perepechaeva M.L., Grishanova A.Y., Rudnitskaya E.A., Kolosova N.G. (2014). The mitochondria-targeted antioxidant SkQ1 downregulates aryl hydrocarbon receptor-dependent genes in the retina of OXYS rats with AMD-like retinopathy. J. Ophthalmol..

[47] Pan H., He M., Liu R., Brecha N.C., Yu A.C., Pu M. (2014). Sulforaphane protects rodent retinas against ischemia-reperfusion injury through activation of the Nrf2/HO-1 antioxidant pathway. PLoS ONE.

[48] Chien J.Y., Chou Y.Y., Ciou J.W., Liu F.Y., Huang S.P. (2021). The effects of two Nrf2 activators, bardoxolone methyl and omaveloxolone, on retinal ganglion cell survival during ischemic optic neuropathy. Antioxidants.

[49] Saito Y., Geisen P., Uppal A., Hartnett M.E. (2007). Inhibition of NAD(P)H oxidase reduces apoptosis and avascular retina in an animal model of retinopathy of prematurity. Mol. Vis..

[50] Wood J.P.M., Chidlow G., Wall G.M., Casson R.J. (2024). N-acetylcysteine amide and di-N-acetylcysteine amide protect retinal cells in culture via an antioxidant action. Exp. Eye Res..

[51] Chronopoulos P., Manicam C., Zadeh J.K., Laspas P., Unkrig J.C., Göbel M.L., Musayeva A., Pfeiffer N., Oelze M., Daiber A. (2023). Effects of resveratrol on vascular function in retinal ischemia-reperfusion injury. Antioxidants.

[52] Shahror R.A., Fouda A.Y. (2025). Recent advances and future challenges in nanosystems for ocular drug delivery. J. Pharmacol. Exp. Ther..

[53] Naguib S., Backstrom J.R., Gil M., Calkins D.J., Rex T.S. (2021). Retinal oxidative stress activates the NRF2/ARE pathway: An early endogenous protective response to ocular hypertension. Redox Biol..

[54] Khayat M., Lois N., Williams M., Stitt A.W. (2017). Animal models of retinal vein occlusion. Investig. Ophthalmol. Vis. Sci..

[55] Wang P., Chin E.K., Almeida D. (2021). Antioxidants for the treatment of retinal disease: Summary of recent evidence. Clin. Ophthalmol..

[56] Hidaka Y., Nakamura S., Nishinaka A., Takajo Y., Inamasu S., Yomoda S., Shimazawa M., Hara H. (2023). Arctigenin prevents retinal edema in a murine retinal vein occlusion model. Biol. Pharm. Bull..

[57] Long P., Yan W., He M., Zhang Q., Wang Z., Li M., Xue J., Chen T., An J., Zhang Z. (2019). Protective effects of hydrogen gas in a rat model of branch retinal vein occlusion via decreasing VEGF-α expression. BMC Ophthalmol..

[58] Darabuş D.M., Dărăbuş R.G., Munteanu M. (2025). The diagnosis and treatment of branch retinal vein occlusions: An update. Biomedicines.

[59] Hatamnejad A., Nanji K., Grad J., El-Sayes A., Mihalache A., Gemae M., Huang R., Waheed N.K., Sarraf D., Sadda S.R. (2026). Predicting treatment response in retinal vein occlusions using baseline optical coherence tomography biomarkers: A systematic review. Surv. Ophthalmol..

[60] Campa C., Alivernini G., Bolletta E., Parodi M.B., Perri P. (2016). Anti-VEGF therapy for retinal vein occlusions. Curr. Drug Targets.

